# Elevated vitreous body glial fibrillary acidic protein in retinal diseases

**DOI:** 10.1007/s00417-015-3127-7

**Published:** 2015-08-18

**Authors:** Anselm Gerhard Maria Jünemann, Robert Rejdak, Cord Huchzermeyer, Ryszard Maciejewski, Pawel Grieb, Friedrich E. Kruse, Eberhart Zrenner, Konrad Rejdak, Axel Petzold

**Affiliations:** Department of Ophthalmology, University of Erlangen-Nurnberg, Erlangen, Germany; Department of General Ophthalmology, Medical University of Lublin, Lublin, Poland; Medical Research Centre, Polish Academy of Science, Warsaw, Poland; Department of Neurology, Medical University of Lublin, Lublin, Poland; Department of Anatomy, Medical University of Lublin, Lublin, Poland; Expertise Center Neuro-ophthalmology, Free University Medical Center, Amsterdam, The Netherlands; Department of Neuro-Ophthalmology, Moorfields Eye Hospital, City Road, London, UK; UCL Institute of Neurology, Queen Square, London, WC1N 3BG UK

**Keywords:** Vitreous, Glial fibrillary acidic protein (GFAP), Biomarker, Retinal detachment, Epiretinal gliosis, Müller cells

## Abstract

**Purpose:**

Increased expression of glial fibrillary acidic protein (GFAP) is a characteristic of gliotic activation (Müller cells and astrocytes) in the retina. This study assessed vitreous body GFAP levels in various forms of retinal pathology.

**Methods:**

This prospective study included 82 patients who underwent vitrectomy (46 retinal detachments (RDs), 13 macular hole (MHs), 15 epiretinal glioses (EGs), 8 organ donors). An established enzyme–linked immunosorbent assay (ELISA, SMI26) was used for quantification of GFAP.

**Results:**

The highest concentration of vitreous body GFAP in organ donors was 20 pg/mL and it was used as the cutoff. A significant proportion of patients suffering from RD (65 %) to EG (53 %) had vitreous body GFAP levels above this cutoff when compared to organ donors (0 %, *p* < 0.0001, *p* = 0.0194, respectively, Fisher’s exact test) and MH (8 %, *p* < 0.0001, *p* = 0.0157, respectively). In RD and EG, vitreous body GFAP levels were correlated with axial length (*R* = 0.69, *R* = 0.52, *p* < 0.05 for both).

**Conclusions:**

The data suggest that human vitreous body GFAP is a protein biomarker for glial activation in response to retinal pathologies. Vitreous body GFAP levels may be of interest as a surrogate outcome for experimental treatment strategies in translational studies.

## Introduction

Glial fibrillary acid protein (GFAP) is a protein biomarker for astrocytes and activated Müller cells [1]. Müller cells are the principle glial cells in the neural retina, being involved in the homoeostasis and metabolism of retinal neurons [2, 3], and they penetrate the entire human retina in radial columns. Astrocytes are only found within the retinal nerve fibre layer (RNFL) and, notably, the optic nerve head (ONH). This protein biomarker is a non–soluble, 432-amino acid cytoskeletal protein belonging to the class–III intermediate filament proteins [1, 4]. Discovered in 1969, it is encoded on chromosome 17q21.1-q25 [1, 5]. Under normal conditions, GFAP is not expressed in Müller cells.

Activation of Müller cells including increased expression of GFAP is a key response feature of the human retina to injury and a sign of age-related subclinical pathologies [6]. It includes morphological, biochemical, and physiological changes in Müller cells and has mixed effects, contributing to neuro*regeneration* including protease activation, but also to neuro*degeneration* with impediment of tissue repair [3]. Müller cells are responsible for scar formation after retinal detachment with devastating consequences for recovery of visual function [6].

The up-regulation of GFAP within the Müller cells is a remarkably ubiquitous response in retinal pathology [7] and the most sensitive non-specific response to retinal disease and injury. Of note, GFAP is not required for normal functioning of Müller cells, only for Müller cell gliosis. Disintegration of Müller cells triggers cellular proteolysis. With proteolytic break–up of the GFAP polymer, soluble fragments of GFAP are released to the adjacent fluid compartments [1, 8]. This process is calcium dependent and calpain-mediated release of a 41 kDa fragment dominates over other, smaller proteolytic break-down products of GFAP [1].

Therefore, GFAP might be used as an indirect marker for retinal injury, Müller cell activation, protease activation and finally secondary degenerative processes in the retina [2, 7]. Notably, there is experimental and clinical evidence for increased GFAP expression in retinal Müller cells in RD [9–14] and proliferative vitreoretinopathy [15, 16]. Furthermore, the intraretinal glial response appears to be centrally involved in the formation of epiretinal gliosis (EG) [7]. Vitreous body proteomics has emerged as a tool to better understand and quantify the cellular processes underlying ocular disease [17, 18]. An advantage of enzyme-linked immunosorbent assay (ELISA) techniques is that the advanced analytical sensitivity allows detection of minute amounts of protein in a high throughput setting [1].

Therefore, we hypothesized that using a sensitive ELISA for GFAP [19] on vitreous body material from patients with RD and EG might permit us to detect GFAP from the human vitreous body, although GFAP has not yet been reported to be present in the vitreous body before. Interestingly, it was even absent in a large-scale proteomic study of patients suffering from a proliferative vitreoretinopathy (PVR), which revealed 97–137 vitreous body proteins [20]. This is surprising because GFAP is such an abundant protein, extremely stable and well suited for mass spectrometry [21]. Of note, the study by Yu et al. detected another intermediate filament, the neurofilament protein, albeit at a very low level. Using a highly sensitive ELISA, we previously described vitreous body neurofilament levels in a range of retinal pathologies [22]. We further hypothesised that, if detectable, vitreous body GFAP levels would be higher in these conditions compared with normal controls or patients with macular holes (MHs), where glial retinal pathology is less dominant.

## Materials and methods

### Patients

This study was approved by the ethics committees of the local clinical centres (Lublin, Poland and Erlangen, Germany) and adhered to the tenets of the Declaration of Helsinki. Acquisition of research samples was limited to routine sampling procedures. As a control, we included vitreous body material from cadaveric ocular tissue.

### Vitreous body homogenate

Vitreous body samples were collected from 74 patients who underwent vitrectomy for RD (*n* = 46), EG (*n* = 15) and MH surgery (*n* = 13). Because it is ethically not possible to obtain vitreous body from healthy controls, we also included organ donors (*n* = 8). All samples were coded, snap–frozen and stored at −80 °C until analysis. On receipt in London, the samples were thawed and homogenised on ice using a Sonipre 150 (power 14, one minute). The homogenate was spun down (4 °C, 150,000 rpm, 10 min) and the supernatant was used for analysis.

### Sample analysis

All samples were analysed with the analyst being blinded to all other data.

A previously described, an ELISA technique was used to quantify vitreous body homogenate levels of GFAP [19]. In brief, microtitre plates were coated overnight with 100 μL of the SMI26 capture antibody and diluted 1/5000 in 0.05-M carbonate buffer, pH 9.5. The plate was washed with barbitone buffer containing 6-mM EDTA, 0.1-% bovine serum albumin BSA and 0.05-% Tween 20 (pH 8.6). The plate was blocked with 250 μL of barbitone buffer containing 6 mM EDTA and 1 % of BSA. After washing, 50 μL of barbitone buffer, 6-mM EDTA, 0.1-% BSA were added as sample diluent to each well. Fifty μL of standard or vitreous body homogenate samples were then added in duplicate to the plate. The plate was incubated at room temperature (RT) for 1 h. After washing, 100 μL of of horseradish peroxidase (HRP)-labelled rabbit anti-bovine GFAP diluted 1:1000 in barbitone buffer (6-mM EDTA, 0.1-% BSA) were added to each well and the plate was incubated for 1 h at RT. After a final wash, 100 μl of tetramethylbenzidine (TMB) substrate were added. The plate was incubated for 20 min at RT in the dark; the reaction was stopped by adding 50 μl 1-M hydrochloric acid (HCl) and the absorbance was read at 450 nm with 750 nm as the reference wavelength on a Wallac Victor 2 ELISA plate reader.

### Data analysis

All statistical analyses and graphs were done using SAS software (version 9.4, SAS Institute, Inc., Cary, North Carolina, USA). Both mean (± standard deviation, SD) and median values were presented because non–Gaussian distribution independent variables were compared using the non-parametric two-sample exact Wilcoxon rank-sum test for two variables and a two–way unbalanced analysis of variance (ANOVA; general linear model (GLM)) for more than two variables. Fisher’s exact test was used for comparing the proportion of patients with pathological (positive, high) GFAP levels because of small sample size and distribution. The cut–off above which results were considered pathological was defined as the highest value observed in the control group. The linear relationship between continuous variables was evaluated using the Spearman correlation coefficient. Multiple correlations were corrected using the Bonferroni method. Linear regression analysis was performed using the least squares method.

## Results

The demographic features of the patients are summarised in Table 1. There was no difference in age between the groups (*F* = 1.40, *p* = 0.3) and there was no correlation between the patients’ age and the vitreous body GFAP levels for either the pooled cohort or any of the diagnostic groups.

**Table 1:** Patients characteristics (mean values ± standard deviation)

Characteristic	OD	EG	MH	RD
Number	8	15	13	46
Age	63 ± 18	70 ± 7	70 ± 6	64 ± 15
Gender (F:M)	4:4	8:7	8:5	14:32
Onset to surgery (days)	–	503 ± 567	116 ± 55.4	97.1 ± 261
VA	–	0.28 ± 0.18	0.18 ± 0.09	0.25 ± 0.27
IOP	–	12.3 ± 2.9	13.0 ± 3.2	11.7 ± 4.4
Axial length [mm]	–	23.7 ± 1.9	22.8 ± 0.8	24.5 ± 2.3
PVR	–	–	–	8/22 (36 %)
MD	–	–	–	15/22 (68 %)
DM	1/8 (13 %)	1/7 (14 %)	1/4 (25 %)	5/21 (24 %)
RR	1/8 (13 %)	4/7 (57 %)	3/5 (60 %)	10/22 (45 %)
GFAP [pg/mL]	11 ± 4	158 ± 302	12 ± 9	241 ± 342

For those variables where the data was incomplete, the total number of patients (%) is shown.

*DM* diabetes mellitus, *IOP* intraocular pressure (mmHg), *MD* macular detachment, *MH* macular hole, *PVR* proliferative vitreoretinopathy, *RD* retinal detachment, *RR* arterial hypertension, *EG* epiretinal gliosis, *MD* macular degeneration, *OD* organ donors, *VA* visual acuity. * = results are significantly different compared to patients with RD

**Fig. 1:**
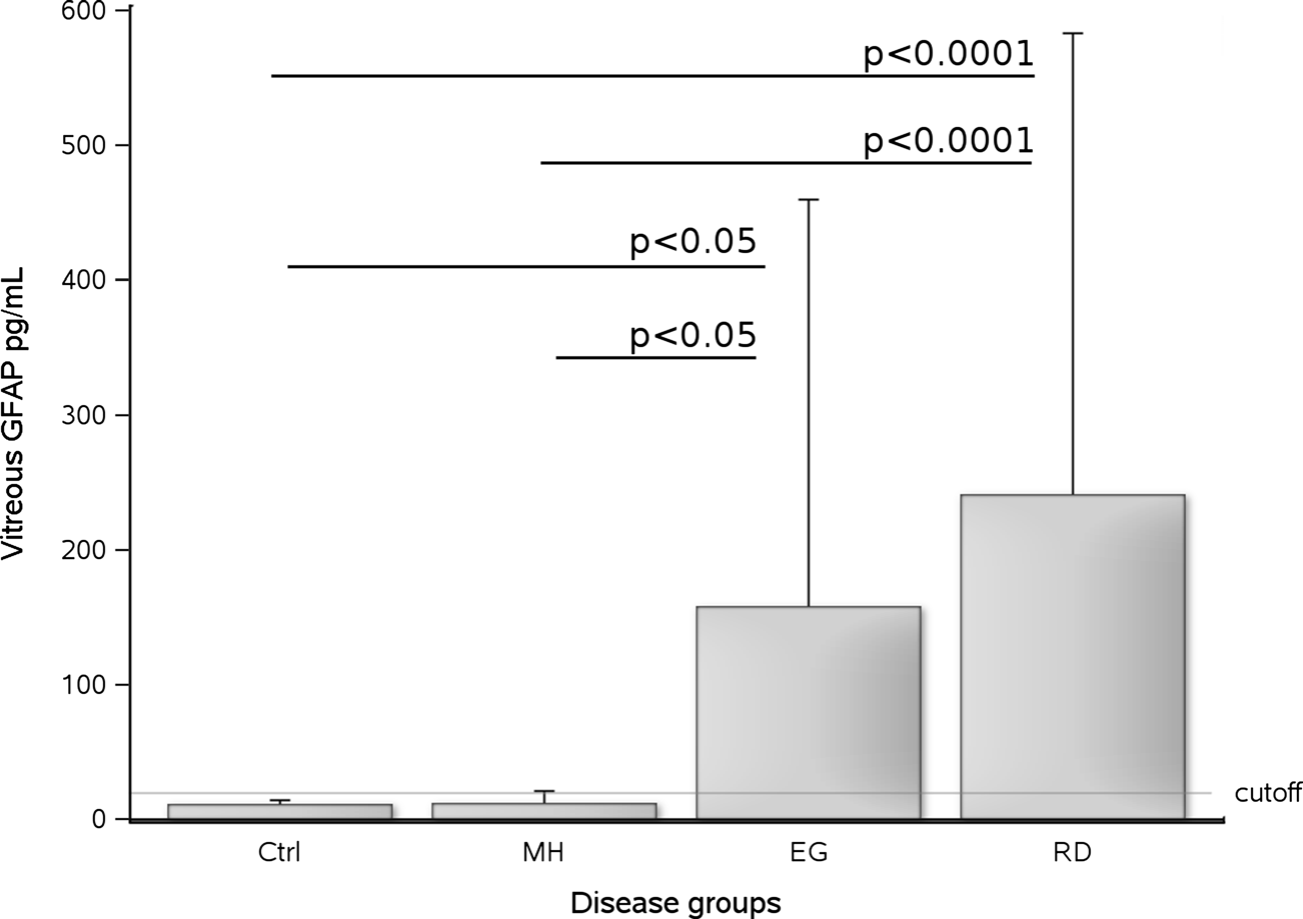
A protein biomarker for glial (Müller cell and astrocytes) damage in retinal disease, vitreous body GFAP. According to the upper reference range (cutoff) of 20 pg/mL (*horizontal reference line*) high vitreous body homogenate GFAP levels were observed in 0/8 (0 %) of organ donors (OD), 1/13 (8 %) of patients with a MH surgery, 8/15 (53 %) of patients with EG and 30/46 (65 %) of patients with retinal detachment (RD). The mean and standard deviation are shown

The highest concentration of GFAP protein found in the vitreous body from the organ donor control group was 20 pg/mL (horizontal *dotted line* in Fig. 1). According to this cut-off, pathological vitreous GFAP levels were found in 8/15 (53 %) of the patients with EG, 1/13 (8 %) patients with MHs and 30/46 (65 %) patients with RD. The proportion of patients with pathological vitreous body GFAP levels were significantly higher following RD when compared to all organ donors (two–sided Fisher’s exact test *p* < 0.0001) and MHs (*p* < 0.0001). Likewise vitreous body GFAP levels were higher in a significantly larger proportion of patients with EG when compared to organ donors (two–sided Fisher’s exact test *p* = 0.0194) and MHs (*p* = 0.0157). There was no significant difference in vitreous body GFAP levels between patients with RD and EG (*p* > 0.05).

Figure 1 shows the averaged GFAP levels (+SD) grouped according to the patients’ diagnosis. The median value of the organ donors (10 pg/mL, range 10–20 pg/mL) was comparable to the median value following MH surgery (median 10, range 0 to 30 pg/mL). Medians were higher in EG (median 30, range 0 to 1100 pg/mL) and RD (70, range 0 to 1100 pg/mL).

Vitreous body GFAP levels were not correlated to the time delay between onset of symptoms and sampling of the vitreous body (*R* = −0.09, *p* = 0.6, data not shown).

**Fig. 2:**
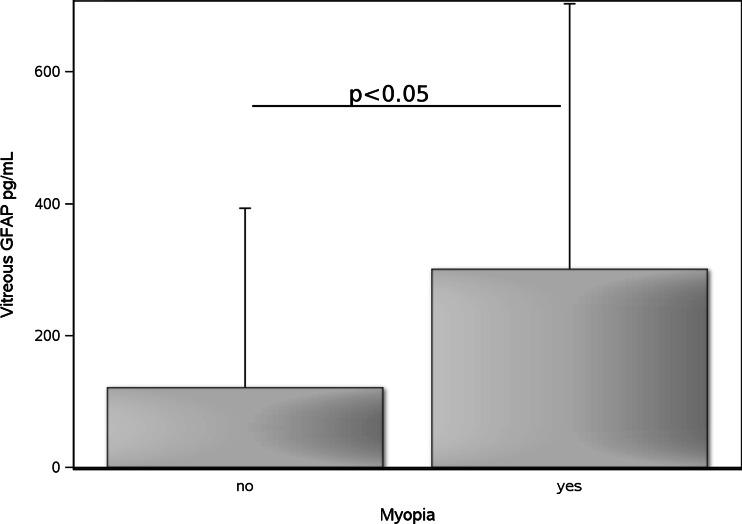
Vitreous body GFAP levels were higher in patients with myopia (*n* = 22) compared to those without (*n* = 27, *p* = 0.024). The mean and SD are shown

Patients with myopia (> −0.5 dpt) had significant higher median vitreous body GFAP levels (50 ng/mL) compared to those with hyperopia (20 pg/mL, *p* = 0.024, Fig. 2). Additionally, the axial length was correlated with vitreous body GFAP levels in the pooled cohort (*R* = 0.59, *p* = 0.0005) as well as in patients with EG (*R* = 0.69, *p* = 0.027) or RD (*R* = 0.52, *p* = 0.039).

## Discussion

In the present study, we found increased levels of GFAP in the vitreous body of patients with RD and EG. The normal range of vitreous body GFAP levels was determined from donor eyes. The normal GFAP levels were below 20 pg/mL. In RD, the GFAP levels in the vitreous were increased 20-fold, and in RG, the increase was 10-fold.

To the best of our knowledge, this is the first study quantifying GFAP levels in the human vitreous. GFAP has not yet been reported to be present in the vitreous body using a different technique [21]. Using a highly sensitive ELISA, we previously described vitreous body neurofilament levels in a range of retinal pathologies [22]. Thus, highly sensitive ELISA seems to be necessary to detect GFAP in the vitreous, too [1, 19].

The degenerative vitreous processes potentially induce pathology at the vitreoretinal interface [23, 24]. In RD, Müller cell hypertrophy and hyperplasia expand into the subretinal space and vitreous, and is associated with GFAP production [9, 25–27].

These unspecific responses of Müller cells start within 1 day of detachment [12, 28, 29]. At 3 days of detachment, the low basal level of GFAP mRNA in the normal retina is increased to approximately 500 % [26] with GFAP labelling of Müller cells extending throughout the retina [30]. Elevated GFAP expression was found in Müller cells in early and long-term RD. Increased GFAP-containing intermediate filaments within Müller cells were found in 30- and 60-day detached retinas, as determined by protein gels, immunoblotting analysis, and light- and electron-microscopic immunocytochemistry [9, 31]. Furthermore, tissue autoradiographic studies indicate an increase in RNA synthesis between 2 and 3 days after RD in Müller cells [32, 33] in parallel with a five-fold increase in GFAP mRNA at that time using RNA blotting analysis and *in situ* hybridization [26].

In the vitreous, further translational studies are needed to address the time course, GFAP levels and outcome after surgical repair, as upregulation of intermediate filaments seems to be a crucial step for the gliotic response [3]. Epiretinal, intraretinal and subretinal reactive gliosis of Müller cells is a clinically significant limiting factor in the recovery of vision after reattachment [14, 34, 35]. It has been proposed that attempts to reduce Müller cell gliosis may inhibit subsequent retinal degeneration and support neuroregeneration after reattachment [14, 35].

Interestingly, myopic eyes showed significant higher median GFAP levels in comparison to hyperopic eyes. Even though the numbers in this study are small, this finding is consistent with the literature on myopia as a relevant eye condition. Myopic eyes show more and severe retinal degeneration, more frequently RD [36] and, generally, the outcome from retinal repair surgery remains poorer [37].

The increased GFAP levels in EG in this study indicate the role of Müller cells in this retinal disease, too [38]. The normal levels of GFAP in MHs might be due to different unspecific or specific responses of Müller cells to the kind of stimulus in MH disease or to a small lesion size.

The limitations to this study are related to the fact that GFAP as a soluble protein biomarker can only provide indirect evidence for retinal pathology. Next, the definition of the cutoff value was based on organ donors, which cannot be regarded as a healthy control group. In addition, the post-mortem interval was not recorded. It is, however, conceivable that artefactual vitreous GFAP levels in this study are unlikely because, technically, the removal of the eye is quicker than the spinal cord where absence of a post-mortem artefact for GFAP has been demonstrated [39]. In addition, the GFAP levels from patients with a MH were in a comparable range to those from the organ donors. Taken together, this argues against a bias introduced by the post-mortem interval of the organ donors in the present study.

This study did not show a correlation between vitreous body GFAP levels and time delay onset of symptoms and sampling. This is likely due to the large time range (Table 1). This range exceeds what is known about the detachment-induced proliferation of Müller cells which peaks within 3 to 4 days [13, 28]. The time lag of an average 91 days between RD to surgery might explain the lack of a time-GFAP correlation. But it might be also due to the relative weak power of historical data regarding the onset of symptoms and the range of RD duration. Other limitations of this study are the small numbers and the lack of follow-up. It would be of interest to discern if GFAP levels at time of surgery are of prognostic value in RD and EG. Extending on this limitation, the heterogeneous delay between symptom onset and sampling prevented us from performing a systematic investigation of RD severity with vitreous GFAP levels. Future studies on the prognostic value of vitreous body GFAP levels should include such severity data so as to enable testing the hypothesis that higher GFAP levels are present with more severe disease. Such studies will be a prerequisite for testing the clinical relevance of this biomarker. Finally, patient numbers were not equally balanced. There were relatively few patients with a MH. Therefore, this study cannot exclude a limited degree of glial activation in MHs as suggested by the literature [40, 41].

In conclusion, we found, for the first time, increased GFAP levels in the vitreous of patients suffering from RD and EG. Because upregulation of GFAP is the most sensitive non-specific response of Müller cells in retinal disease, vitreous GFAP levels might act as a biomarker for retinal glial activation. GFAP levels in the vitreous were also elevated in cases of myopia. The data suggest that human vitreous body GFAP may be an interesting protein biomarker for indirect quantification of the glial response to retinal disorders. The small sample volume required for the ELISA can be further reduced using electrochemiluminescence- or fluorescence-based methods [1]. This opens avenues for future translational studies on the cell culture and animal models using vitreous body GFAP levels as a surrogate outcome.
